# Effects of Zn Deficiency and Bicarbonate on the Growth and Photosynthetic Characteristics of Four Plant Species

**DOI:** 10.1371/journal.pone.0169812

**Published:** 2017-01-11

**Authors:** Kuan Zhao, Yanyou Wu

**Affiliations:** 1 School of Environment and Resources, Anqing Normal University, Anqing, P. R. China; 2 Key Laboratory of Modern Agricultural Equipment and Technology, Ministry of Education, Jiangsu University, Zhenjiang, P. R. China; 3 State Key Laboratory of Environmental Geochemistry, Institute of Geochemistry, Chinese Academy of Sciences, Guiyang, P. R. China; Huazhong University of Science and Technology, CHINA

## Abstract

Calcareous soils are characterized by low nutrient contents, high bicarbonate (HCO_3_^−^) content, and high alkalinity. The effects of HCO_3_^−^ addition under zinc-sufficient (+Zn) and zinc-deficient (−Zn) conditions on the growth and photosynthetic characteristics of seedlings of two Moraceae species (*Broussonetia papyrifera* and *Morus alba*) and two Brassicaceae species (*Orychophragmus violaceus* and *Brassica napus*) were investigated. These four species were hydroponically grown in nutrient solution with 0 mM Zn (−Zn) or 0.02 mM Zn (+Zn) and 0 mM or 10 mM HCO_3_^−^. The photosynthetic response to HCO_3_^−^ treatment, Zn deficiency, or both varied according to plant species. Of the four species, *Broussonetia papyrifera* showed the best adaptability to Zn deficiency for both the 0 mM and 10 mM HCO_3_^−^ treatments due to its strong growth and minimal inhibition of photosynthesis and photosystem II (PS II). *Brassica napus* was sensitive to Zn deficiency, HCO_3_^−^ treatment, or both as evidenced by the considerable inhibition of photosynthesis and high PS II activity. The results indicated different responses of various plant species to Zn deficiency and excess HCO_3_^−^. *Broussonetia papyrifera* was shown to have potential as a pioneer species in karst regions.

## Introduction

Bicarbonate (HCO_3_^−^) is the product for the catalysis of carbon dioxide (CO_2_) hydration by carbonic anhydrase (CA). It can be used as an inorganic carbon source to supplement CO_2_ in leaf cells [1]. Additionally, HCO_3_^−^ is an essential constituent of the water-oxidizing complex of photosystem II (PS II). This complex is stabilized by HCO_3_^−^ by binding to other components of PS II and influences the molecular processes associated with the electron acceptor and electron donor sides of PS II [1,2]. Finally, HCO_3_^−^ supplies CO_2_ and H_2_O through the actions of CA in photosynthetic oxygen evolution under environmental stress [3]. However, excess HCO_3_^−^ is harmful for crop growth due to the inhibition of protein synthesis and respiration and decreased nutrient absorption [4].

Zinc (Zn) is an essential microelement for plant growth in all kinds of soils. It influences many biological processes, including carbohydrate metabolism, cell proliferation and phosphorus-Zn interactions [5–7]. Zn also serves as an integral component of some enzyme structures, such as CA, alcohol dehydrogenase, and glutamate dehydrogenase [7]. Therefore, Zn deficiency causes the rapid inhibition of plant growth and development, which results in increased reactive oxygen species (ROS) due to photo-oxidative damage and consequently decreased net photosynthesis and photosynthetic electron transport [8].

Excess HCO_3_^−^ or Zn deficiency inhibits photosynthesis and PS II, which influences photosynthetic and chlorophyll (Chl) fluorescence parameters [9–11]. HCO_3_^−^, which is considered the key factor that influences Fe deficiency chlorosis and Zn deficiency in many plant species [12], is the major anion found in calcareous soils in karst regions. However, few studies have shown how plant growth and photosynthetic physiology react to the dual impact of Zn stress and excess HCO_3_^−^. The response to excess HCO_3_^−^ in different rice genotypes has indicated that Zn-efficient rice cultivars can sustain root growth in the presence of high HCO_3_^−^ when grown in soils with low Zn availability, whereas root growth in Zn-inefficient genotypes is severely inhibited [13,14].

Karst regions are characterized by calcareous soils with a low bioavailability of plant nutrients (e.g., phosphorus, Zn, and iron), high calcium content (in the form of calcium carbonate), and high alkalinity (pH 7.5 to 8.5) [15]. Zn deficiency in plants is particularly associated with calcareous soils in karst regions, where the HCO_3_^−^ concentration in surface runoff water is approximately 5 mM [16]. HCO_3_^−^ is considered an important factor for inhibiting plant growth in calcareous soil, especially in rice and wheat [17]. To grow in these challenging regions, plants must adapt and overcome the prevalent nutrient deficiency in these soils. In a previous study, nine calcifuge and nine acidifuge plants exuded different organic acids from their roots when grown in calcareous soils [18]. Wu and Xing [3] demonstrated that *Broussonetia papyrifera* (L.) Vent. and *Morus alba* L. can alternatively absorb CO_2_ from the atmosphere and HCO_3_^−^ under excess HCO_3_^−^ stress. The plants utilized HCO_3_^−^ in the form of converted CO_2_ and water, and the conversion was reversibly catalyzed by CA. Thus, different plant species have different physiological response modes that allow them to adapt to environments with low Zn and excess HCO_3_^−^.

*B*. *papyrifera* and *M*. *alba*, which belong to the Moraceae family, are characterized by a rapid growth rate and greater adaptability to low-nutrient and excess-HCO_3_^−^ environments than other members of the family [19–21]. *Orychophragmus violaceus* L. *Schulz* and *Brassica napus* L. are members of the Brassicaceae family that can grow in karst environments. However, *O*. *violaceus* is better than *B*. *napus* at accumulating available nutrients from the rhizosphere [22]. Therefore, the different growth environment of *B*. *papyrifera*, *M*. *alba*, *O*. *violaceus* and *B*.*napus* influenced difference of adaptability response.

Our previous research focus on the biomass, the physiological and biochemical property such as carbonic anhydrase activity, photosynthesis of *Broussonetia papyrifera* in Karst soils [19]. As the complexity and universality of Karst soil, whether Zn deficiency or excess bicarbonate is the main factor influencing bicarbonate-use capacity and plant growth was hypothesized under hydroponics. In this study, plant growth and the characteristics of both photosynthetic and Chl fluorescence of four species (*Broussonetia papyrifera*, *Bp*; *Morus alba*, *Ma*; *Orychophragmus violaceus*, *Ov*; *Brassica napus*, *Bn*) were investigated under Zn-deficient and excess HCO_3_^−^ conditions. Furthermore, the effects of Zn stress and HCO_3_^−^ treatment on the photosynthetic characteristics of the four plant species were analyzed, and the mechanisms underlying various adaptive responses of different plant species were investigated.

## Materials and Methods

### Plant culture and experimental treatments

Seeds of the four plant species (two Moraceae plants: *Broussonetia papyrifera*, *Bp*; *Morus alba*, *Ma*; and two Brassicaceae plants: *Orychophragmus violaceus*, *Ov*; *Brassica napus*, *Bn*) were surface sterilized (5 min in 95% ethanol and 30 min in 10% H_2_O_2_ with a wash in sterile water after each treatment), sown and grown for 15 days in plastic pots filled with normal Hoagland nutrient solution. Then, the seedlings were transferred into modified Hoagland nutrient solution containing (mM) KNO_3_, 5.0; Ca(NO_3_)_2_∙4H_2_O, 4.0; NH_4_NO_3_, 1.0; KH_2_PO_4_, 0.25; MgSO_4_∙7H_2_O, 1.0; H_3_BO_3_, 0.05; MnSO_4_∙4H_2_O, 0.004; CuSO_4_∙5H_2_O, 0.005; Fe(Na)EDTA, 0.03; and (NH_4_)_6_Mo_7_O_24_∙4H_2_O, 0.002 with no Zn (Zn-deficient, −Zn) or 0.02 mM ZnSO_4_∙7H_2_O (Zn-sufficient, +Zn) combined with no HCO_3_^−^ (0) or 10 mM (10) HCO_3_^−^. The pH was adjusted to 8.0 using 1 M KOH before HCO_3_^−^ was added to the nutrient solution. The four treatments were named +Zn0 (adequate Zn and no HCO_3_^−^), +Zn10 (adequate Zn and 10 mM HCO_3_^−^ addition), −Zn0 (Zn deficiency and no HCO_3_^−^), and −Zn10 (Zn deficiency and 10 mM HCO_3_^−^ addition). HCO_3_^−^ was supplied as sodium bicarbonate. The plants were grown in a controlled environment with a photosynthetic photon flux density (PPFD) of 300 μmol m^−2^ s^−1^, a 14-h photoperiod, a temperature of 25 ± 0.5°C and a relative humidity of 55 ± 2%. During the experiments, the solution was changed every two days. Measurements were conducted in duplicate on day 15.

### Photosynthetic parameter measurements

Photosynthetic parameters, such as net photosynthetic rate (*P*_N_), transpiration (E), stomatal conductance (*g*_*s*_), and intercellular CO_2_ concentration (Ci), were measured using a portable LI-6400XT photosynthesis system (LI-COR Inc., Lincoln, NE, USA). The fourth-youngest fully expanded leaf from the top was used for measurement between 9:00 and 11:00 a.m. The photosynthetic active radiation, temperature, and CO_2_ concentration during measurement collection were 600 μmol m^−2^ s^−1^, 25°C, and 380 μmol mol^−1^, respectively. The water use efficiency (WUE) was calculated as *P*_N_/E.

### Chl fluorescence measurements

Chl fluorescence parameters were measured using an IMAGING-PAM Chl fluorometer (Heinz Walz GmbH, Effeltrich, Germany) that applied the same array of blue light-emitting diodes (peak wavelength, 470 nm) for fluorescence excitation, actinic illumination, and saturating light pulses. Plants were dark-adapted for 30 min prior to measurement using the upper middle fully expanded leaves [23]. The minimum Chl fluorescence (Fo) was determined using a measuring beam, whereas the maximum Chl fluorescence (Fm) was measured during an 800-ms exposure to a saturating light intensity (6000 μmol m^−2^ s^−1^). The Fm′ (maximal fluorescence yield of a light-adapted leaf) and steady-state Chl fluorescence (Ft) were determined. The maximum quantum yield of PS II (Fv/Fm) was calculated as (Fm − Fo)/Fm. The effective PS II quantum yield (ΦPS II) was calculated as (Fm′ − Ft)/Fm′. Therefore, the relative photosynthetic electron transport rate (ETR) was calculated as ΦPS II × PPFD × 0.5 × 0.84.

### Chl content and biomass measurements

The Chl content was determined for the 3^rd^ and 6^th^ fully expanded leaves (in two leaf-age stages) counted from the top of the plants (three measurements per leaf) using SPAD-502 readings (Konica Minolta Sensing Inc., Osaka, Japan). Each measurement was repeated five times. Leaf, stem, and root samples of plants from each of the four treatments were dried at 105°C for 30 min and then weighed at 70°C to obtain their dry weight (DW).

### Zinc concentration measurement in roots, stems and leaves

The dried samples of roots, stems and leaves were digested with HNO_3_–HClO_4_, and the Zn concentrations in the plants were determined using a TAS-990 hydride-flame atomic absorption spectrometer (Persee Inc., Beijing, China).

### Determination of the variation in growth and photosynthetic characteristics

To compare the plant response to Zn stress and HCO_3_^−^ treatment, variations in plant biomass, photosynthetic parameters, Chl fluorescence parameters, and Chl content were calculated according to Eqs (1–4).

$$A_{1}=\frac{\left(G_{+\text{Zn}10}-G_{+\text{Zn}0}\right)}{G_{+\text{Zn}0}}\times 100\%$$ (1)

$$A_{2}=\frac{\left(G_{\text{-Zn}10}-G_{\text{-Zn}0}\right)}{G_{\text{-Zn}0}}\times 100\%$$ (2)

$$A_{3}=\frac{\left(G_{\text{-Zn}0}-G_{+\text{Zn}0}\right)}{G_{+\text{Zn}0}}\times 100\%$$ (3)

$$A_{4}=\frac{\left(G_{\text{-Zn}10}-G_{+\text{Zn}10}\right)}{G_{+\text{Zn}10}}\times 100\%$$ (4)

where *G* is the plant biomass, photosynthetic parameters, Chl fluorescence parameters, Chl content, or Zn concentration of organs under Zn stress and/or HCO_3_^−^ treatment; *A*_1_ is the influence of HCO_3_^−^ treatment under +Zn conditions, and the control is the +Zn0 treatment (Eq (1)); *A*_2_ is the influence of HCO_3_^−^ treatment under −Zn conditions, and the control is the −Zn0 treatment (Eq (2)); *A*_3_ is the influence of Zn deficiency under no HCO_3_^−^ conditions, and the control is the +Zn0 treatment (Eq (3)); and *A*_4_ is the influence of Zn deficiency under HCO_3_^−^ treatment, and the control is the +Zn10 treatment (Eq (4)). The unit of *A*_*i*_ (*i* = 1, 2, 3, 4) is percentage; positive values indicate stimulation, whereas negative values indicate depression.

The plant resistance to HCO_3_^−^ addition or Zn deficiency according to the *A*_*i*_ value was assessed from the biomass, Chl content, photosynthetic parameters (*P*_N_, WUE, *g*_*s*_), Chl fluorescence parameters (ETR, Fv/Fm) and Zn concentration of the organs.

## Results

### Plant biomass and Chl content

**Fig 1:**
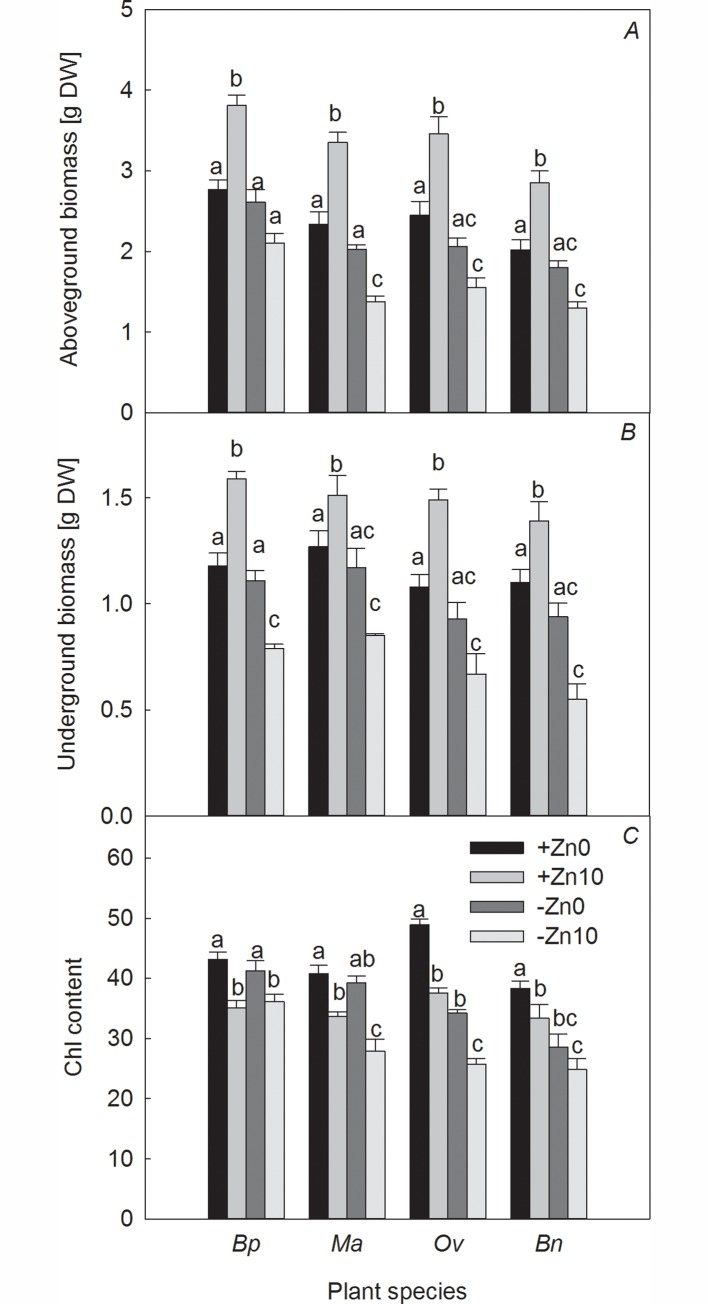
Effects of Zn and HCO_3_^−^ on the biomass and Chl content of the four plant species. Note: Columns with bars indicate the mean ± SE (n = 5). Lowercase letters indicate a significant difference among the four treatments for the same plant species at *p* < 0.05.

Under +Zn conditions, the biomass of all four plant species increased with the presence of HCO_3_^−^ (+Zn10 treatment) (Fig 1*A* and 1*B*). The addition of HCO_3_^−^ inhibited the Chl content in all four plant species (Fig 1*C*). Compared to those under +Zn0 treatments, *Ma* and *Bp* showed maximum and minimum increases in aboveground (underground) biomass of 43.16% (37.96%) and 37.55% (18.90%), respectively. As observed in Fig 1, *Ov* showed the greatest decrease in Chl content.

Under −Zn conditions, the biomass of all four plant species decreased with HCO_3_^−^ addition (−Zn10 treatment) (Fig 1*A* and 1*B*). Compared to the −Zn0 treatment, HCO_3_^−^ addition had the largest adverse effect on the aboveground biomass of *Ma* and the underground biomass of *Bn*, as well as the smallest adverse effect on *Bp* biomass. The decreases in the aboveground biomass of *Ma*, the underground biomass of *Bn* and the aboveground (underground) biomass of *Bp* were 32.02%, 41.49% and 19.54% (27.35%), respectively (Fig 1).

Under no-HCO_3_^−^ conditions, Zn deficiency had an adverse effect on the biomass and Chl content in all four plant species. Compared to those in the +Zn0 treatments, the decreases in the aboveground biomass of *Bp*, *Ma*, *Ov* and *Bn* were 5.78%, 13.25%, 15.92% and 10.89%, respectively. The Chl content of the two Brassicaceae plants decreased more significantly than in the two Moraceae species (Fig 1).

Under HCO_3_^−^-treatment conditions, Zn deficiency had a significant inhibitory effect on biomass, which had the greatest inhibitory effect on the aboveground biomass of *Ma* and the underground biomass of *Bn* (Fig 1*A* and 1*B*). Compared to those in the +Zn10 treatment, the decreases in *Ma* in the aboveground biomass and *Bn* in the underground biomass were 58.81% and 60.43%, respectively. The Chl content decreased much more in the two Brassicaceae plants than in the two Moraceae species. *Bp* showed a small increase in Chl content (Fig 1).

### Chl fluorescence and photosynthetic parameters

**Fig 2:**
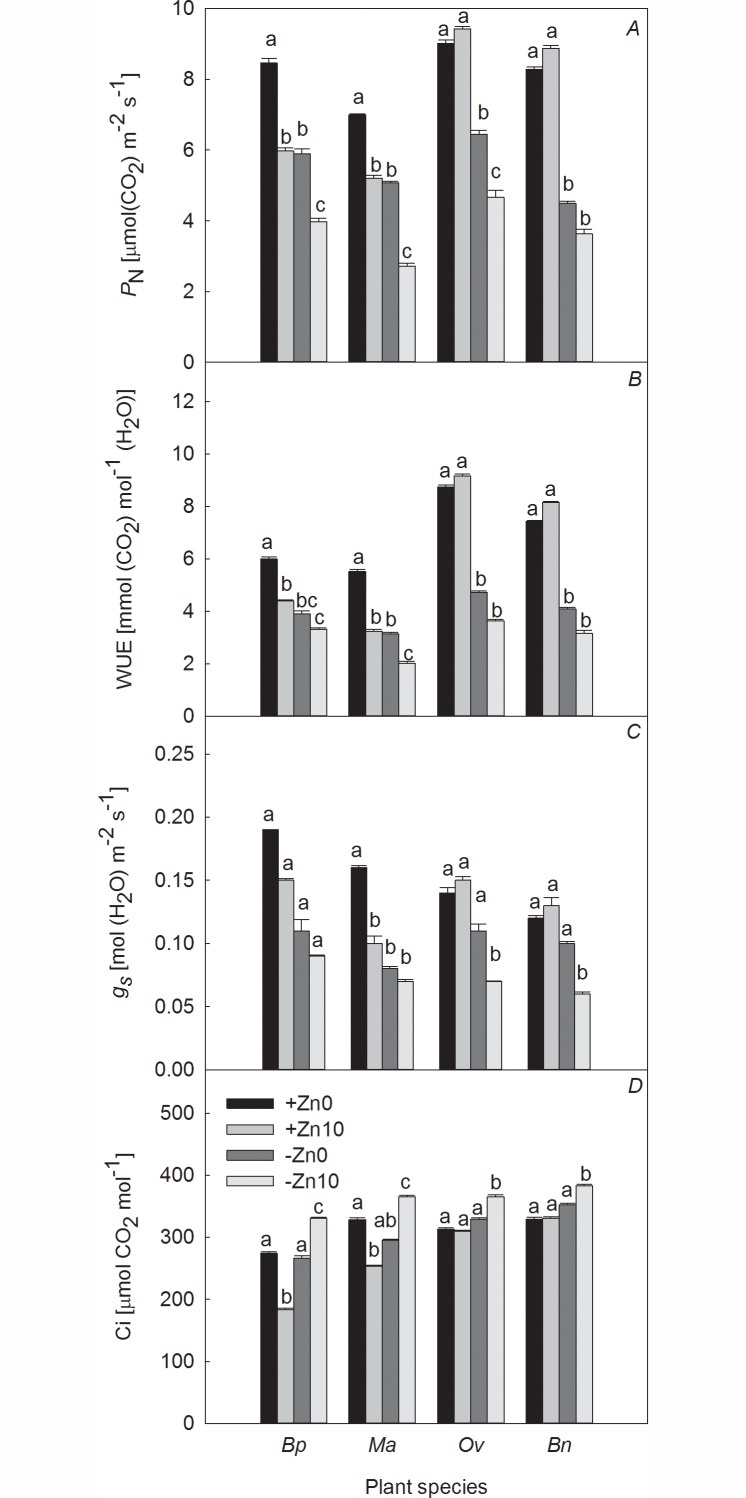
Effects of Zn and HCO_3_^−^ on the photosynthetic parameters of the four plant species. Note: Columns with bars indicate the mean ± SE (n = 5). Lowercase letters indicate a significant difference among the four treatments for the same plant species at *p* < 0.05.

**Fig 3:**
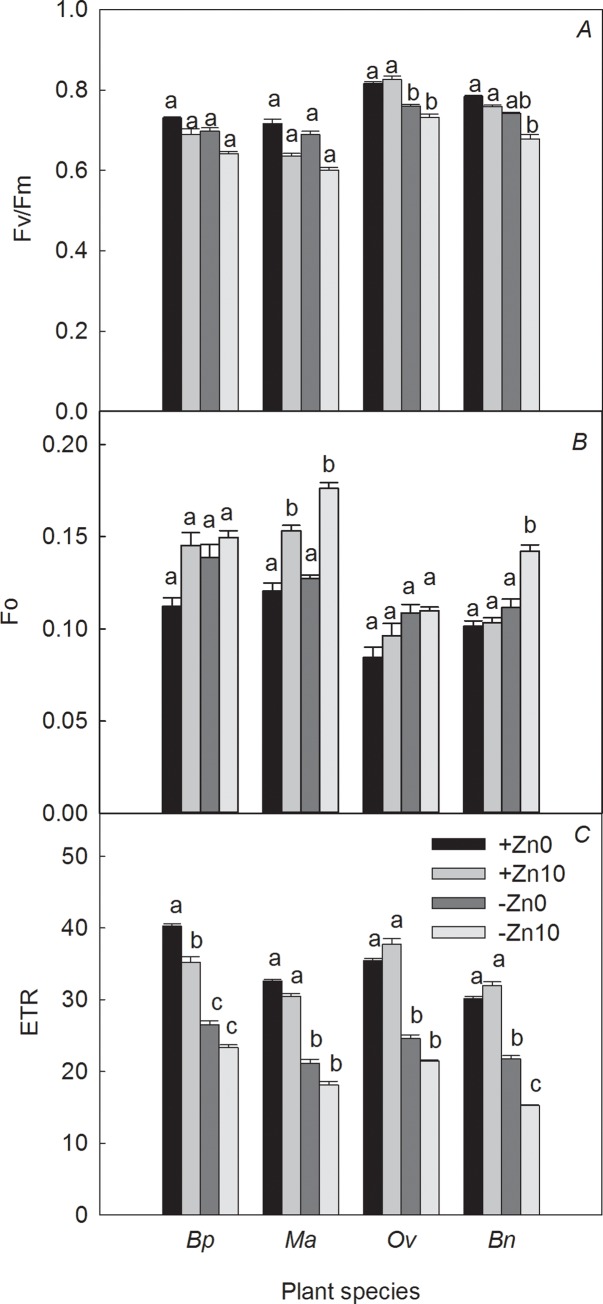
Effects of Zn and HCO_3_^−^ on the Chl fluorescence parameters of the four plant species. Note: Columns with bars indicate the mean ± SE (n = 5). Lowercase letters indicate a significant difference among the four treatments for the same plant species at *p* < 0.05.

Under +Zn conditions, HCO_3_^−^ addition increased Fo and decreased *P*_N_, *g*_*s*_, Fv/Fm, and ETR. Additionally, it had an adverse effect on the WUE in both Moraceae species. Compared to the +Zn0 treatment, *Ma* showed the greatest decrease in WUE (41.20%), whereas *P*_N_, WUE, *g*_*s*_, Fv/Fm, and ETR increased in both Brassicaceae species, except for the Fv/Fm of *Bn* and the Fo of *Ov* (Figs 2 and 3).

Under −Zn conditions, the *P*_N_ and WUE of all species significantly decreased with the addition of HCO_3_^−^. Fv/Fm was also slightly inhibited, and Fo increased remarkably in *Ma* and *Bn*. Compared with the −Zn0 treatments, HCO_3_^−^ addition produced the greatest decrease in WUE in *Ma* and the smallest decrease in WUE in *Bp*, i.e., 35.46% and 14.87%, respectively. The increases in the Fo of *Bp*, *Ma*, *Ov* and *Bn* were 7.90%, 38.58%, 6.05% and 27.48%, respectively (Figs 2 and 3).

Under no-HCO_3_^−^ conditions, Zn deficiency inhibited *P*_N_ and ETR in all four plant species and had a small adverse effect on Fv/Fm. Compared with the +Zn0 treatments, Zn deficiency led to the maximum inhibition of *P*_N_ in *Bn* (45.65%) and the minimum inhibition of WUE in *Bp* (35.00%). The Fo in *Bp* and *Ov* was greater than that in *Ma* and *Bn*. The increases in the Fo of *Bp*, *Ma*, *Ov* and *Bn* were 23.56%, 5.46%, 22.53% and 10.13%, respectively (Figs 2 and 3).

Under HCO_3_^−^-treatment conditions, Zn deficiency decreased *P*_N_ and increased Ci. It also had a small influence on Fv/Fm and dramatically inhibited ETR in all four plant species, in addition to significantly increasing the Fo in *Ma*. Compared with the +Zn10 treatment, Zn deficiency increased the Fo and decreased the *P*_N_, WUE, *g*_*s*_, Fv/Fm, and ETR in the Brassicaceae plants more severely than in the two Moraceae species. The decreases in the P_N_, WUE, Ci, and ETR of *Bp* were the smallest, while those parameters of *Bn* were the greatest under the dual action of HCO_3_^−^ and low Zn (−Zn10 treatment). The decreases in the *P*_N_, WUE and ETR of *Bp* were 33.50%, 24.55% and 33.68%, respectively, while those of *Bn* were 59.08%, 61.23% and 52.31%, respectively. The inhibition of growth, photosynthesis, and electron transport under the interaction of Zn and HCO_3_^−^ exceeded that under Zn deficiency or HCO_3_^−^ treatment (Figs 2 and 3).

### Zn concentration in plant organs

As shown in Table 1, the Zn concentration in four plant organs significantly decreased with Zn deficiency and excess HCO_3_^−^. The Zn concentration in the organs of two Moraceae plants was significantly higher than that in the two Brassicaceae plants.

**Table 1:** Effects of Zn and HCO_3_^−^ on the Zn concentration (mg Kg^-1^ DW) in the roots, stems and leaves of the four plant species. Note: Values are the mean (M) ± standard error (SE) (n = 5). Different lowercase letters indicate a significant difference between the four treatments for the same plant organs at *p* < 0.05; different capital letters indicate a significant difference in organs of the same species under the same treatment at *p* < 0.05.

Plant species	Organs	Zn and HCO_3_^−^ treatments			
		+Zn0	+Zn10	−Zn0	−Zn10
*B*. *papyrifera*	Roots	54.74aA ± 1.28	42.33bA ± 1.93	30.17cA ± 0.98	23.67dA ± 0.82
	Stems	62.78aB ± 1.67	45.72bAB ± 1.87	32.73cA ± 1.01	20.23dB ± 0.84
	Leaves	88.33aC ± 2.70	50.27bB ± 2.26	36.77cB ± 1.50	19.79dB ± 0.56
*M*. *alba*	Roots	50.64aA ± 1.10	41.17bA ± 2.55	28.10cA ± 1.23	18.26dA ± 1.01
	Stems	57.94aB ± 1.38	38.56bAB ± 2.34	29.76cA ± 1.21	15.17dB ± 0.78
	Leaves	74.4aC ± 1.50	45.73bB ± 1.90	27.68cA ± 1.09	15.22dB ± 0.73
*O*. *violaceus*	Roots	23.22aA ± 1.17	24.67aA ± 1.34	21.86aA ± 0.79	14.06bA ± 1.13
	Stems	24.72aA ± 1.09	26.28aA ± 1.57	14.90bB ± 0.62	9.73cB ± 0.87
	Leaves	32.66aB ± 2.04	30.26aB ± 1.29	17.72bC ± 1.31	11.28cB ± 1.04
*B*. *napus*	Roots	24.35aA ± 1.29	20.18aA ± 1.57	18.40aA ± 0.77	11.17bA ± 1.09
	Stems	30.34aB ± 2.37	29.17aB ± 1.38	16.00bA ± 0.53	9.74cB ± 0.83
	Leaves	34.73aC ± 2.55	28.72aB ± 1.89	15.11bB ± 0.62	8.78cB ± 0.69

Under +Zn conditions, the Zn concentration of organs from all four plant species decreased in the presence of HCO_3_^−^ (+Zn10 treatment), except for that of the roots and stems of *Ov*. There was a significantly greater decrease in Zn concentration in the organs of the two Moraceae plants than in those of the two Brassicaceae plants. The largest Zn decrease occurred in the leaves of all four plant species, and the increases in the leaves of *Bp*, *Ma*, *Ov* and *Bp* were -43.09%, -38.53%, -7.35% and -17.30%, respectively.

Under −Zn conditions, the Zn concentration of all four plant species organs decreased with HCO_3_^−^ addition (−Zn10 treatment), and the decrease in the Zn concentration in the aboveground parts (leaves and stems) was greater than that in the underground parts of the two Moraceae plants. There were no significant differences between the aboveground parts and underground parts of the two Brassicaceae plants.

Under no-HCO_3_^−^ conditions, Zn deficiency significantly decreased the Zn concentration in the two Moraceae plants. The Zn concentration in the leaves showed the greatest decrease in all four plant species. The decreases in the leaves of *Bp*, *Ma*, *Ov* and *Bp* were 58.37%, 62.80%, 45.74% and 56.49%, respectively.

Under HCO_3_^−^-treatment conditions, the greatest decrease in Zn concentration in all four plant species occurred due to the interaction of Zn deficiency and excess HCO_3_^−^, and the Zn concentration decrease in the underground parts (roots) in all four species was greater than 45%, particularly in the roots of *Ma*, where the Zn concentration decreased by as much as 55.65%. The Zn concentration decrease in the aboveground parts (leaves and stems) of all four species was greater than 55%, particularly in the leaves and stems of *Bn*, where the Zn concentration decreased by as much as 69.71% and 66.61%, respectively (Table 1).

## Discussion

### Response of plant growth to excess HCO_3_^−^ and/or Zn deficiency

**Fig 4:**
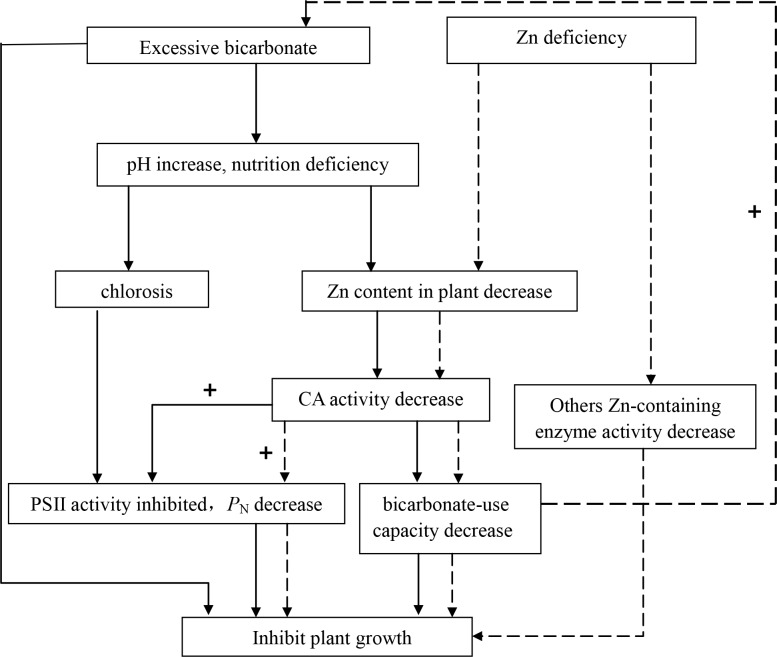
The response of plant growth to excess HCO_3_^−^ and/or Zn deficiency. Note: solid-line arrows indicate the mechanism of excess HCO_3_^−^ on plant growth; dotted-line arrows indicate the mechanism of Zn deficiency on plant growth. CA: carbonic anhydrase. *P*_N_: net photosynthetic rate. +: homonymous stimulation effects. PS II: photosystem II.

As shown in Fig 4, excess HCO_3_^−^ influenced plant growth; excess HCO_3_^−^ results in an intracellular ion charge imbalance through passive diffusion, thus directly inhibiting plant growth [2]. It also inhibited plant growth indirectly through increased pH, which led to a decrease in available nutrient elements, such as Zn, iron, and copper, and then inhibited plant growth *via* two pathways. In the first pathway, excess HCO_3_^−^ initially appeared in all plants as chlorosis related to nutrient element deficiency [24], a considerable decrease of photosynthetic capability was associated with the lower Zn contents [25], followed by the inhibition of PS II activity and decreased photosynthetic parameters, such as *P*_N_ and WUE, and Chl fluorescence parameters, such as Fo, Fv/Fm, and ETR. In the second pathway, excess HCO_3_^−^ decreased the Zn concentration in plant tissue and the CA activity involved in photosynthetic carbon metabolism, in addition to inhibiting the HCO_3_^−^-use capacity because the substrates provided by HCO_3_^−^ alleviated the CA-catalytic conversion reaction. This effect may be strengthened by the toxicity of HCO_3_^−^. Eventually, CA activity decreased, inhibiting PS II activity and reducing inorganic carbon assimilation under excess HCO_3_^−^ and/or Zn deficiency. In contrast, when the HCO_3_^−^-use capacity increased, the toxicity of HCO_3_^−^ decreased [3,26].

Zn deficiency decreased the Zn concentration in plant tissue and then inhibited the activity of CA, a Zn-containing enzyme that catalyzes the reversible reaction between CO_2_ hydration and HCO_3_^−^ dehydration (Fig 4). Low CA activity resulted in a slight conversion of HCO_3_^−^ into CO_2_ and H_2_O under Zn deficiency [27,28]. As such, Zn deficiency inhibited the plant HCO_3_^−^-use capacity and PS II activity due to the presence of excess HCO_3_^−^. Meanwhile, Zn deficiency inhibited other Zn-containing enzymes, such as alcohol dehydrogenase and glutamate dehydrogenase, thus inhibiting plant growth [29].

### Plant resistance to excess HCO_3_^−^ and/or Zn deficiency

Different responses arose from different modes of photosynthetic response to excess HCO_3_^−^ and/or Zn deficiency in various plant species. The resistance of each plant to excess HCO_3_^−^ or Zn deficiency was different, as shown in Table 2. The total photosynthetic assimilation of inorganic carbon, including CO_2_ and HCO_3_^−^, in *Bp* under excess HCO_3_^−^ might exceed its total photosynthetic assimilation without the addition of HCO_3_^−^. In addition, the activity of CA in *Bp* was about 2 times higher than that in *Ma* on either an sunny days or a cloudy days in Karst soils [19]. *Bp* had a significantly greater CA activity, at least five times greater than that in *Ma* under 10 mM bicarbonate treatments in hydroponically culture [3]. High concentrations of bicarbonate decreased the photosynthetic assimilation of inorganic carbon in *Bp* and *Ma* in none bicarbonate treaments [3]. Therefore, the growth of *Bp* increased under these conditions. *Bp* had a greater HCO_3_^−^-use capacity than did *Ma* [3,26]. Although there was a decrease in *P*_N_ in *Bp*, the Zn concentration was greater than that in *Ma*, and the growth of *Bp* was stimulated because of the considerable HCO_3_^−^-use capacity under excess HCO_3_^−^ conditions. The conclusion is identical with our previous research conclusions in the field soil cultivations a higher CA activity of *Bp* supplied both water and CO_2_ for the photosynthesis of mesophyll cells [3,19]. However, we illuminated Zn deficiency or excess bicarbonate, or both as the crucial factors influenced bicarbonate-use capacity, the photosynthetic response to excess HCO_3_^−^ or Zn deficiency varied with plant species, and then the difference in plant resistance to nutrition stress.

**Table 2:** Resistance to excess HCO_3_^−^ treatment or Zn deficiency under Zn and HCO_3_^−^ interaction. Note: The criterion index was expressed by # in terms of A*i*: #, very sensitive; # #, sensitive; # # #, weak; # # # #, medium; and # # # # #, strong. Under excess HCO_3_^−^, the A*i* of biomass, P_*N*_ (WUE, *g*_*s*_) and ETR (Fv/Fm, Chl content) were more than 20%, 0 and 0, respectively, when the criterion index was # # # # #; 5~20%, -15~0%, and -10~0%, respectively, when the criterion index was # # # #; -15~5%, -30~-15%, and -20~-10%, respectively, when the criterion index was # # #; -30~-15%, -30~-45%, and -30~-20%, respectively, when the criterion index was # #; and less than -30%, -45% and -30%, respectively, when the criterion index was #. Under Zn deficiency, the Ai of biomass, P_*N*_ (WUE, ETR, Fv/Fm) and Chl content were more than -10%, -15% and 0 when the criterion index was # # # # #; -20~-10%, -25~-15%, and -10~0, respectively, when the criterion index was # # # #; -30~-20%, -35~-25%, and -20~-10%, respectively, when the criterion index was # # #; -40~-30%, -45~-35%, and -30~-20%, respectively, when the criterion index was # #; and -40%, -45% and -30%, respectively, when the criterion index was #.

Plant species	HCO_3_^−^ treatment		Zn deficiency	
	Zn-sufficient	Zn-deficient	No HCO_3_^−^	HCO_3_^−^ treatment
*Bp*	# # # #	# # # #	# # # #	# # #
*Ma*	# # # # #	# # #	# # #	# #
*Ov*	# # # # #	# # #	# # #	# #
*Bn*	# # # # #	# #	# # #	#

WUE was correlated with the HCO_3_^−^-use capacity and was also influenced by the photosynthetic rate and inorganic carbon-use capacity [30]. The smallest decrease in the WUE in *Bp* indicated that *Bp* had a greater capacity for HCO_3_^−^ use than did the other three species, and the Zn concentration in the organs of *Bp* was the highest among the four species. Thus, *Bp* showed the smallest decrease in growth, and the organ activity of *Bp* was higher than that in the other three species. Although the *P*_N_ of *Bn* slightly decreased, the PS II ETR, *g*_*s*_ and Zn availability in *Bn* organs were severely inhibited. *Bn* also did not adapt well to excess HCO_3_^−^ under Zn deficiency. As a result, growth, particularly root growth, was inhibited. Thus, *Bp* showed the greatest capacity of all four plant species to resist excess HCO_3_^−^ under Zn deficiency (Table 2).

Few substrates compensate for photosynthesis under the dual influence of Zn deficiency and HCO_3_^−^ treatment. Therefore, the interaction of Zn deficiency and HCO_3_^−^ treatment (−Zn10 treatment) severely inhibited growth, photosynthesis, Zn accumulation in plant organs and electron transport. The photosynthesis of *Bp* was least affected, and the Zn concentration in the *Bp* organs was the highest, indicating that *Bp* might have a greater capacity for inorganic carbon use and resistance to Zn deficiency than the other species that were tested. *Bp* also grew the most rapidly. *Bn* had the greatest inhibition in photosynthesis and PS II reaction center activity, indicating that it had the weakest resistance to Zn deficiency and HCO_3_^−^ addition (−Zn10 treatment) (Table 2).

## Conclusions

Four plant species showed different photosynthetic responses to Zn deficiency, HCO_3_^−^ treatment, or both. *Bp* showed the greatest adaptability to HCO_3_^−^ treatment, Zn deficiency, or both, which involved the greatest HCO_3_^−^-use capacity. *Bn* was sensitive to Zn deficiency, HCO_3_^−^ treatment, or both, due to the great inhibition of photosynthesis and PS II reaction center activity. In summary, the plants had different adaptive modes in response to Zn deficiency, HCO_3_^−^ treatment, or both. According to this research and the previous studies we suggested that *Bp* has the potential to be a pioneer species for ecological restoration in environments with Zn deficiency and excess HCO_3_^–^, such as karst regions.

## Supporting Information

S1 Table The original data of plant biomass, Zn concentration, chlorophyll contents and chlorophyll fluorescence and photosynthetic parameters of two plants. (XLS)
